# Effects of three prophylactic interventions on French middle-schoolers’ mental health: protocol for a randomized controlled trial

**DOI:** 10.1186/s40359-024-01723-8

**Published:** 2024-04-13

**Authors:** Eugénie Vaillant-Coindard, Gaëtan Briet, Florence Lespiau, Béatrice Gisclard, Elodie Charbonnier

**Affiliations:** 1 UNIV. NIMES, APSY-V, F-30021 Nîmes Cedex 1, France; 2 UNIV. NIMES, PROJEKT, F-30021 Nîmes Cedex 1, France

**Keywords:** Adolescents, Anxiety, Depression, Prevention, Promotion, Wellbeing, Cognitive-behavioral therapy

## Abstract

Adolescence is a strategic developmental stage in terms of preventing later difficulties and ensuring good mental health. Prophylactic interventions, which are conducted before the onset, prolongation, or worsening of difficulties, and aim to prevent or reduce symptoms or to promote wellbeing, therefore appear particularly appropriate for adolescents. However, existing prophylactic interventions conducted with adolescents have several weaknesses, including sparse theoretical frameworks, ambivalent evidence of their efficacy, and implementation and dissemination difficulties. In addition, no data are currently available on the effectiveness of such interventions in France. To fill this gap, a four-arm randomized controlled trial will be performed to assess the effectiveness of three prophylactic interventions targeting reactive, proactive and interpersonal adaptation in fourth-grade middle-school students, together with participants’ experience and perception of the interventions. Based on existing knowledge about adolescents, their learning mechanisms, and field constraints, these three interventions have been designed to promote their learning and receptiveness to interventions. Compared with baseline (i.e., before the intervention), we expect to observe a significant decrease in the level of distress (anxiety and depressive symptoms, functional impairment, and psychosocial difficulties) and a significant increase in the level of wellbeing after the intervention, across the three intervention groups, but not in the control group. In addition, we expect to observe post-intervention improvements in the processes targeted by the reactive adaptation intervention (operationalized as coping strategy use and flexibility), those targeted by the proactive adaptation intervention (operationalized as the tendency to engage in committed actions and general self-efficacy), and those targeted by the interpersonal adaptation intervention (operationalized as assertiveness in interactions), but only in the corresponding groups, with no change in any of these processes in the control group. The results of this research will not only enrich our knowledge of the processes involved in adolescents’ distress and wellbeing, but also provide clues as to the best targets for intervention. Moreover, the material for these interventions will be freely available in French on request to the corresponding author, providing access to innovative and fully assessed interventions aimed at promoting adolescents’ mental health in France.

This clinical trial is currently being registered under no. 2023-A01973-42 on https://ansm.sante.fr/. This is the first version of the protocol.

**Supplementary Information:**

The online version contains supplementary material available at 10.1186/s40359-024-01723-8.

## Introduction

Usually lasting from ages 11 years to 19 years [231], adolescence is commonly regarded as a key period characterized by important developmental changes (e.g., physical, cognitive, affective, social, contextual) and tasks (e.g., autonomy, identity, socialization, [37, 50, 51]). These developmental issues critically interact with mental health, in terms of both psychopathology and wellbeing [173, 198, 201]. Half of the most common lifetime mental disorders emerge before the age of 18 years, and more than one third between the ages of 11 and 15 years ([113] for the American population; [196] at the international level), with emergence peaking at 14.5 years [196]. Longitudinal studies indicate that more than four out of ten individuals [94] will exhibit at least one of the most frequent mental disorders at some point during their adolescent years [167]. In addition, the prevalence of mental disorders in adolescence has been estimated to be 10% ([94] for Ireland; [215] worldwide), and even 30% ([20] for the Italian population; [210] for the Danish population; [60] at the international level). Moreover, young people are even more likely to exhibit subclinical symptoms and significant psychological distress [11, 43, 124]. Although no national epidemiological data have yet been gathered in France using reliable diagnostic tools, the available information suggests that 15% of French youth aged 10–20 years would need mental health care [54]. Additionally, recent data indicate that around 23% of 11- to 15-year-old adolescents report moderate to high anxiety or depressive symptoms [178]. It is important to note that last grades-middle-school students (aged about 13–15 years) seem to display a particular clinical fragility, with a third of third and fourth graders being at risk for depression, and higher various psychological symptoms [126]. In a similar vein, in France, adolescents aged 11–14 years displayed the greatest increases in distress and mental health care demand following the COVID-19 pandemic [56].

Wellbeing is an important dimension of mental health [103, 115, 179], and seems to be low to moderate among about one third of adolescents (e.g., [115, 234]), with decreasing trends across adolescence (e.g., [27, 41, 87]). These trends have been monitored worldwide (e.g., [11, 215, 230]), the same ones have been observed in France, with one in three adolescents reporting low-to-moderate levels of wellbeing [70, 126]. Echoing the levels of reported mental health problems, lower levels of wellbeing are observed at the end of middle school than at the beginning [126].

This is all the more alarming given that high levels of distress and low levels of wellbeing in adolescence can increase the risk of psychological issues for adolescent and/or adults, in terms of persistence or increased future severity (e.g., [17, 31, 161]), other psychopathological syndromes or disorders [40, 141], and lower wellbeing [187]. Psychological difficulties in adolescence can be associated with both current and later functional impairment (e.g., [67, 175, 205, 207]), and altered general development [100, 193, 207]. Nevertheless, some studies have identified a wide range of possible trajectories, including both unfavorable and favorable outcomes (e.g., [44, 158],Yang et al., in press). All these factors point to the need to focus on adolescents to promote the development of positive mental health trajectories [53], and, for this purpose, to identify strategic factors influencing mental health trajectories.

Process-based models in developmental psychopathology [89, 154, 217], extended to cover wellbeing development [48, 71], may have a positive influence on these trajectories. These models underline the central role played by cognitive, behavioral, affective and interpersonal proximal processes in mental health issues [71, 88, 154], above and beyond the numerous risk and protective factors [16]. Although research among adolescents is very recent, a growing body of studies have investigated the processes that may be involved in adolescent psychopathology and wellbeing. Research has highlighted three interrelated process areas that appear relevant in adolescents’ mental health, namely reactive adaptation (or coping; e.g., [241]), proactive adaptation (e.g., [97, 99, 116]), and interpersonal adaptation (e.g., [26, 202]).

*Reactive adaptation* implies adequate appraisal by individuals of their control over internal or external stressful agents (i.e., appraised as significant and potentially exceeding their resources), and the automatic and effortful strategies they implement to cope with these agent and their effects [21, 80]. Regarding maturational changes, adolescence is conducive and favorable to the development of reactive strategies (e.g., acquisition of cognitive strategies, specialization in strategy use, better coordination of strategies and contextual relevance; [74, 90, 242]). Nevertheless, new challenges and stressors arising from individual and contextual changes can also complicate, and even undermine, the development of reactive adaptation during adolescence [92, 242]. Reactive adaptation in adolescents has been repeatedly linked to various forms of psychopathology when impaired (e.g., [57, 105, 241]), and to higher levels of wellbeing when adequate (e.g., [58, 136, 222]). Reactive adaptation impairments have also been found to predict clinical difficulties [119, 174], and appear to mediate the effects of distal risk factors (i.e., risk and protective factors indirectly influencing mental health outcomes through the effect of proximal factors, be they socio-economic, familial, or biological; [88, 89]) on adolescent psychopathology (e.g., [6, 36, 238]), and wellbeing (e.g., [33, 188, 233]), whether the studies are cross-sectional or longitudinal [49, 77].

Above and beyond the way they react to events and stressors, adolescents are increasingly regarded as agents of their development. In line with this notion, *proactive adaptation* refers to automatic or effortful strategies displayed by individuals to achieve personally relevant goals [21, 190]. Such goals can be defined as “representations of desired states”, directing behavior toward these states [66], p121) in order to avoid aversive effects and draw in positive effects [95, 96, 199], and are related to self-development and self-realization [21, 189]. Proactive adaptation relies on the abilities to define and hierarchize realistic valued goals (e.g., “It’s important to me to succeed in athletics, more than being the best at video games”; [30, 39, 93]), plan and carry out relevant actions, and to persist in spite of obstacles (e.g., planning to prepare for a competition by performing training and strengthening exercises, effectively practicing despite distractors, and repeating and obtaining extra tutoring in the event of failure [85, 93, 157],). It also requires individuals to assess their progress and flexibly adapt their goal-pursuit strategies (e.g., “Am I better at athletics than before? Do I need to find other ways to make progress?”), including goal reformulation or revision when needed (e.g., extra efforts in sports or history to enhance grades [30, 73, 157],). Sufficient self-efficacy to pursue the goal (i.e., perceived ability to perform the actions, produce expected outcomes, and reach the goal) also appears essential at every step (e.g.,“I am able to carry out the exercises and training, and this will effectively help me to improve my performance”; [12, 135, 194]). Although there is a dearth of research on the specificity of proactive adaptation development in adolescence, adolescents are known to acquire increasing autonomy and latitude for decision-making in their choices and behaviors in various areas of life [135], in contrast to the other-regulation by adults they experienced in infancy [50]. Moreover, cognitive and affective maturation gradually allows for greater integration of long-term concerns, personal values, and intrinsic goals in adolescence [242], underlining the importance of developing adequate proactive adaptation skills to avoid negative effects and obtain positive ones. In line with several theories (e.g., cybernetic theory, [66],Hexaflex model, [93]), various dimensions of adolescents’ proactive adaptation (e.g., goal pursuit, self-efficacy) appear to be linked to diverse forms of psychopathology when impaired (e.g., [168, 177, 221]), and to wellbeing level (e.g., [61, 206, 211]). Proactive adaptation also seems to longitudinally predict mental health outcomes [218] and to mediate the effects of distal risk factors on mental health indicators (e.g., [5, 129, 147]), highlighting a direct influence of proactive processes on adolescent mental health.

Finally, above and beyond intrapersonal processes, individuals permanently interact with a complex social environment that is, particularly influential in adolescence and for mental health outcomes. Hence, *interpersonal adaptation* encompasses social skills (e.g., accurate social cognition, assertive communication, constructive conflict resolution; [28, 42]), social performance (i.e., adequate behavior in social interactions, both reactive and spontaneous, engaging social skills [42, 118],), and social adjustment (i.e., good quality relationships with others in the individual’s various social spheres [42, 91, 184],). Adolescence is characterized by high stakes [4, 166, 201] and the development with respect to socialization and interpersonal abilities (e.g., more complex social cognition and social networks, [19, 118, 166]). Meanwhile, a vast body of literature stresses the importance of social adjustment, behaviors and skills in adolescence for both psychopathology (e.g., [35, 97, 98, 121]) and wellbeing [14, 182, 197]. Clear evidence for direct and predictive relations between interpersonal adaptation and adolescents’ mental health has also come from mediational [86, 117, 142] and longitudinal [14, 63, 127, 153] studies.

Overall, these elements highlight the importance of developing adequate reactive, proactive and interpersonal adaptation throughout the adolescent years, in order to promote a favorable state of mental health. These processes should therefore be preferential targets for interventions with adolescents, especially since evidence suggests that they can be positively modified following relatively short interventions among vulnerable or general-population adolescents (e.g., [22, 24, 82, 140],M. [159, 165]). Cognitive behavioral therapy (CBT)-inspired techniques are potentially suitable for targeting the processes of interest in prophylactic interventions promoting positive mental health trajectories among adolescents, given the evidence of CBT efficacy in both therapeutic [152] and preventive [47, 170, 237] settings. In clinical and developmental psychology, interventions that are implemented prior to the emergence, prolongation, or aggravation of difficulties, in order to prevent or reduce symptoms, promote wellbeing [183], or both, can be labeled as *prophylactic* [169]. To date, prophylactic interventions aimed at adolescents have yielded encouraging results in terms of reducing symptoms (e.g., [69, 134, 186, 204, 208]) and increasing wellbeing (e.g., [134, 162, 181]). Furthermore, these interventions appear to be more effective in middle-schoolers than in high-schoolers [220]. Given the large proportion of the population attending school, as well as the considerable amount of time adolescents spend in schools, the latter offer the ideal setting in which to carry out prophylactic interventions [76, 133, 148]. This is also in line with the clinical staging model for intervention, which emphasizes the need to promote school-based universal programs for improving and/or reinforcing adolescent mental health [191, 192]. These priorities have far wider implications for public health worldwide (e.g., [75, 215, 227–229]), as has recently become quite obvious in France (e.g., [54, 144–146, 148]).

Nevertheless, five main limitations have been identified in the literature. First, interventions targeting both psychopathology and wellbeing are still scarce. Second, despite increasing efforts to implement them in schools, there are major constraints in terms of limited temporal, financial and human resources. Third, the efficacy of interventions is still tempered by small effect sizes, reducing the reliability of results obtained thus far [131, 160, 224]. Fourth, few studies have simultaneously investigated the effects of interventions on both indicators of mental health, and targeted processes. Fifth and lastly, despite increasing interest in implementing workshops intended to promote socioemotional learning in French schools, there is still a considerable lack of fully assessed interventions that are designed to proactively enhance adolescents’ mental health. In addition, the latter is still less frequently targeted in preventive interventions among middle-schoolers [104], while despite their strong empirical support, CBT techniques are still poorly integrated into the field of prophylaxis in France.

To sum up, there is a major need for the development of interventions that can effectively improve adolescent trajectories and are easy to implement in schools. This is especially true in France, where no intervention with the explicit primary goal of targeting both psychopathology and wellbeing has yet been assessed. Consequently, given the contributions and limitations of previous research, the present study will seek to assess the effectiveness of three interventions targeting reactive adaptation, proactive adaptation, and interpersonal adaptation, as well as looking at user experience. The study will take the form of a randomized controlled trial to ensure good quality methodology, conducted in a naturalistic setting to enhance ecological validity. Concerning effectiveness, when comparing levels of participants’ processual and mental health outcomes before and after the interventions, we expect to observe improvements in each of the three experimental groups, illustrated by increased levels of wellbeing and decreased levels of distress, functional impairment, and psychosocial difficulties. We predict there will be no improvements in the control group. Furthermore, we expect to observe improvements in 1) the processes targeted by the reactive adaptation intervention (operationalized as coping strategy use and flexibility), 2) those targeted by the proactive adaptation intervention (operationalized as a tendency to engage in committed actions and general self-efficacy), and 3) and those targeted by the interpersonal adaptation intervention (operationalized as assertiveness in interactions), but only in the corresponding groups. We predict that there will be no changes in these processes would in the control group. The middle-term maintenance of intervention effects will also be assessed at 3-month follow-up. Regarding user experience, we predict that participants in the intervention groups will report high levels of satisfaction at the end of each session and following the intervention, in terms of perceived acceptability (or desirability), utility, usability and general appreciation. These measures will also be collected from the control group and compared with those of participants in the intervention groups from an exploratory perspective, and as a controlled variable in comparison analyses.

## Method and analyses

### Participants

Participants will be fourth-grade students (aged about 13 years). Aside from clinical and interventional relevance [126, 220], choice of fourth graders has been motivated by implementation concerns, as third grade is dedicated to national exams in France (middle-school certificate), leading to a tight schedule for students and teachers, with less room for the interventions.

We plan to include between 120 (37% exclusion rate) and 190 participants to increase statistical power, as a minimum of 100 students was recommended by a G*Power^©^ analysis, based on an alpha risk of 0.05, 95% power, a 5% margin error, and expected minimum effect sizes of 0.19 [225]. Inclusion criteria will be as follows: enrolled in participating schools, fourth-grade students, consent of one of their parents or legal guardians to participate, assent from the students themselves to participate in the study, and proficiency in the French language for both parents and students. Exclusion criteria will be as follows: refusal to participate from adolescent or parent/legal guardian, and absence from two or more sessions.

### Material and procedure

#### Sociodemographic measures

Student age and gender will be collected from adolescents. The following sociodemographic data will be collected from parents (or legal guardians): adolescent’s past or current somatic and psychiatric diagnosis, previous and current pharmacological and psychotherapeutic treatments, and living arrangements (e.g., with one parent or both), each parent’s gender, age, occupation, education level, and marital status, mean household income, and information about siblings.

#### Processual outcomes

Reactive adaptation will be assessed with the French version [149] of the dispositional Brief-COPE Inventory [38], and the French version [212] of the Coping Flexibility Scale [108]. The Brief-COPE comprises 28 items investigating 14 coping strategies (i.e., active coping, planning, positive reappraisal, acceptance, denial, feelings expression, blame, humor, religion, distraction, substance use, behavioral disengagement, and instrumental and emotional support-seeking), corresponding to strategy subscales. Participants rate each item on a Likert scale ranging from 1 (*Not at all*) to 4 (*Always*). Each strategy subscale yields a specific strategy score (ranging from 2 to 8), which, when high, indicates a frequent strategy use. The Coping Flexibility Scale comprises seven items measuring the perceived effectiveness of coping strategies (3 items), as well as the participant’s ability to use more adaptive strategies if previous strategies were ineffectual (4 items). Participants rate each item on a 4-point Likert scale ranging from 1 (*Not applicable to me*) to 4 (*Fully applicable to me*), with an overall score ranging from 7 to 28 which, when high, indicates good coping flexibility. The French versions of these two tools both have good psychometric properties, in terms of reliability and validity in young people [149, 212].

Proactive adaptation will be measured with a French adaptation of the Action subscale of the Willingness and Acceptance Measure for Children and Adolescents (WAM-C/a; [52]), and the short French version [64] of the General Self-Efficacy Scale (S-GSES; [18]). The WAM-C/a’s Action subscale comprises nine items and measures participants’ tendency to take actions in the direction of valued goals (e.g., ‘I do things that are important to me, no matter how I feel’; ‘I try to achieve my goals no matter what’). Participants rate each item on a 5-point Likert scale ranging from 0 (*Not true at all*) to 4 (*Very true*), yielding a subscale score ranging from 0 to 36, with a high score indicating a high tendency to take valued actions despite negative feelings. The WAM-C/a items were translated into French by one of the investigators, and then back into the original language (Spanish) by several bilingual speakers. The S-GSES includes three items measuring participants’ confidence in their ability to achieve tasks and handle difficulties (e.g., ‘In difficult situations, I can rely on my aptitudes’; ‘I can overcome most of my problems on my own’). Participants rate each item on a 5-point Likert scale ranging from 1 (*Doesn’t suit me at all*) to 5 (*Fully suits me*), yielding an overall score ranging from 5 to 15, where a high score indicates a high level of self-efficacy. Both the original Spanish version of the WAM-C/a [52] and the French version of the S-GSES [64] have displayed satisfactory psychometric properties in adolescents.

Interpersonal adaptation will be assessed with a French adaptation of the Assertiveness Formative Questionnaire [155]. This 20-item scale (six reverse-scored items) measures participants' appraisal of their tendency to assertively express wants, needs, and thoughts (e.g., ‘I express my opinions, even if others disagree with me’; ‘I often have a hard time saying “No”’, reverse-scored item) and to respect others (e.g., in disagreements: ‘I am careful to avoid hurting other people's feelings, even when I feel that I have been wronged’; ‘In disagreements, I make sure that I understand other points of view’). Items are rated on a 5-point Likert scale ranging from 1 *(Not very like me*) to 5 (*Very like me*) and yield an overall score comprised between 20 and 100, with a high score indicating elevated assertiveness. The original version has shown acceptable reliability and indicators of validity in adolescents [83]. The questionnaire was translated into French by one of the investigators, and then back into the original language (English) by several bilingual speakers.

#### Mental health outcomes

##### Psychopathology

*Anxiety-related and depressive symptom*s [112] will be assessed with the French version [25] of the 14-item self-report Hospital Anxiety and Depression Scale [240], which has shown adequate reliability and factor structure in adolescents [226]. Seven items assess anxiety-related symptoms (e.g., ‘I feel tense or “wound up”’, ‘Worrying thoughts go through my mind’), and seven others assess depressive symptoms (e.g., ‘I feel as if I am slowed down’, ‘I have lost interest in my appearance’), with items from the two subscales interleaved. Scales range from 0 to 3 for each item, with six reversed items. Scores range from 0 to 21 for each subscale, with higher scores reflecting higher levels of anxiety or depressive symptoms.

*Functional impairments*, regarded as a transdiagnostic marker of psychological difficulties (DSM-V, [3]), will be measured with a French adaptation of the self- and parent-report Work and Social Adjustment Scale for Youth [62, 106].This five-item scale measures students’ functional impairments stemming from their thoughts, feelings, or behaviors in various areas of life (school work, household chores, leisure time alone or with others, social relationships), including items such as ‘Some of my thoughts, feelings, or behaviors prevent me from doing well at school / enjoying free time spent alone / forming and maintaining close relationships with other people, including those I live with’). Participants rate each item on an 9-point Likert scale ranging from 0 (*Not at all impaired*) to 8 (*Very severely impaired*), yielding an overall score of 0–40, with a high score indicating a higher level of impairment. The original scale has shown acceptable consistency and validity in middle adolescents [62].

*Psychosocial functioning* will be assessed with the French version (from Massachusetts General Hospital website) of the parent-report Pediatric Symptoms Checklist [107]. This 35-item scale measures young people’s psychosocial difficulties observed in daily life in school or social functioning, in terms of risky behavior or evident emotional symptoms, with items such as ‘[My child] spends more time alone / fights with other children / has trouble with sleeping / has school grades dropping’. Parents rate each item on a 3-point Likert scale ranging from 0 (*Never*) to 2 (*Often*), yielding an overall social functioning score that ranges from 0 to 70, with a high score indicating considerable psychosocial difficulties in daily life functioning. The original version has shown acceptable psychometric properties in youth [107, 151], and relevance when used to assess middle-school adolescents [150].

##### Wellbeing

Wellbeing will be measured with the French version [68] of the self-report Mental Health Continuum – Short Form [123]. This 14-item scale measures adolescents’ wellbeing based on Keyes’ conceptualization [114]. It assesses three dimensions of wellbeing, namely emotional (e.g., ‘During the past month, how often did you feel happy ?’), psychological (e.g., ‘[…] that your life has a sense of direction or meaning to it’), and social (e.g., ‘[…] that you belonged to a community, like a social group, school, neighborhood, etc.’) wellbeing, through three subscales (three, six, and five items). Items are rated on a 6-point scale ranging from 0 (*Never*) to 5 (*Always*), and yields an overall score ranging from 0 to 70 as well as three subscores (0–15 for emotional wellbeing, 0–30 for psychological wellbeing, and 0–25 for social wellbeing), with a high score indicating a high level of wellbeing. This scale has shown appropriate psychometric properties with adolescents [68, 172].

Measures that have been translated or adapted, in the absence of a French validation, can be provided by the investigators on request.

#### Measures related to user experience

User experience will be investigated in three populations (i.e., students, parents, and students’ head teachers), through several theoretical constructs [213, 214]. Four indicators will be considered: perceived acceptability, perceived utility, perceived usability, and general appreciation. *Perceived acceptability* corresponds to the ability of an intervention to meet participants’ needs, goals, expectations, and characteristics, as well as local contingencies such as contextual constraints. This dimension will be assessed through items such as ‘Interventions on students’ wellbeing are part of a school’s mission / worth devoting school time to’. *Perceived utility* measures the extent to which users think that the interventions are beneficial with respect to their goals. Utility will be measured both before and after the intervention, through items such as ‘Interventions this type are useful / necessary’. *Perceived usability* refers to ease with which the components of an intervention can be used (in the case of aids, media, workbooks, other materials), carried out (in the case of activities), and appropriated. This dimension will be measured among students only, through items such as ‘I have understood what we learnt during the workshops’, ‘I feel able to use what we have learned in my daily life’. Finally, a general appreciation of the intervention will be investigated through items such as ‘I am glad that my class is participating (participated)’, or ‘I enjoyed the aids / activities’. Questionnaires will be administered before the beginning of the intervention, to assess users’ baseline perception of it (7–12 items rated on a 5-point Likert scale), and after the first three sessions, to assess users’ immediate experience of the intervention (8–36 items rated on a 5-point Likert scale, plus blank spaces to collect opinions). The assessment will be repeated 3 months after the end of the intervention, to assess users’ delayed perceptions of the it (30 items rated on a 5-point Likert scale, and blank spaces to collect opinions; students only), and their experience related to its content.

Moreover, a short user experience questionnaire (adherence check), comprising nine items rated on a 5-point Likert scale ranging from 0 (*adjective*) to 4 (*opposite adjective*), will be submitted to students at the end of each session, to assess their perceptions of specific session content and conduct. It will explore general appreciation (e.g., ‘I really (dis)liked the workshop’), utility (e.g., ‘I found the workshop not at all useful / very useful’), and usability (‘I didn’t understand anything / understood everything’). This measure will also include items probing students’ cognitive engagement in the activities (e.g., ‘I was very distracted / focused’) to ascertain how they processed information related to the session’s content [110].

Further details about the user experience measures are available in Supplementary Materials 1.

### Interventions

#### Rationale for intervention’ design

A preliminary study has already been conducted, to create sessions based on the needs of last grades middle-schoolers. It was inspired by a social design approach that is an increasingly integrated part of intervention development [130]. This approach postulates that end users must play an active role in the development stage of a product or service [79], to optimize its appropriateness and its match with beneficiaries and context of use [1, 9]. More specifically, our modus operandi was based on a research-project approach to social innovation design, understood as design for social benefit [46], relying on users’ abilities [132], and taking into account the way users experience and act in their ecological context [156]. This participatory approach, intended to build bridges between scientific research and field practices (and between *top-down* and *bottom-up* scientific approaches) in order to improve the relevance of the product or service [81], involves experiments (informed by theoretical inputs) and its co-design with users [78]. Hence, interviews, observations, and ecological testing, conducted by students enrolled on a Masters’ program in social innovation design at [removed for anonymous reviewing] University, were used to create an initial version of the set of tools used in the various sessions (e.g., use of boards, allowing for group discussion and handwriting). This version was improved by the research team so that it better integrates theory-based mental health elements and CBT techniques. In this process, particular care was taken to ensure developmentally relevant usability and attractiveness, resulting in fewer material elements, aged-adapted vocabulary and word games for naming key tools, and colorful, simple and schematized media. A test–retest method involving simulations and role playing was also used to improve sessions and material, making if possible, for example, to detect and adapt unclear instructions, superfluous information, or poorly intelligible content.

Insights from cognitive and education sciences were also taken on board, to ensure that the interventions would effectively promote learning (see below). Hence, sessions were developed in order to 1) provide knowledge related to adaptive processes strategies [219], 2) enhance participants’ ability to recognize a situation as relevant for strategy use (i.e., fostering transfer; [59, 84, 102]), 3) support the inhibition of irrelevant automatic strategies [29, 101], e.g., under the influence of emotional factors; [2], 4) promote metacognitive regulation of the strategies used [59, 219], and 5) reinforce participants’ positive and realistic beliefs about their ability to use relevant strategies [219]. At the theoretical level, the design rationale therefore involved 1) formal elicitation of procedural knowledge about adequate strategy mobilization and metacognitive regulation, and 2) formal training, refinement and automatization of this procedure and of strategies’ mobilization and evaluation.

#### Intervention structure and delivering

##### Intervention structure

**Fig. 1:**
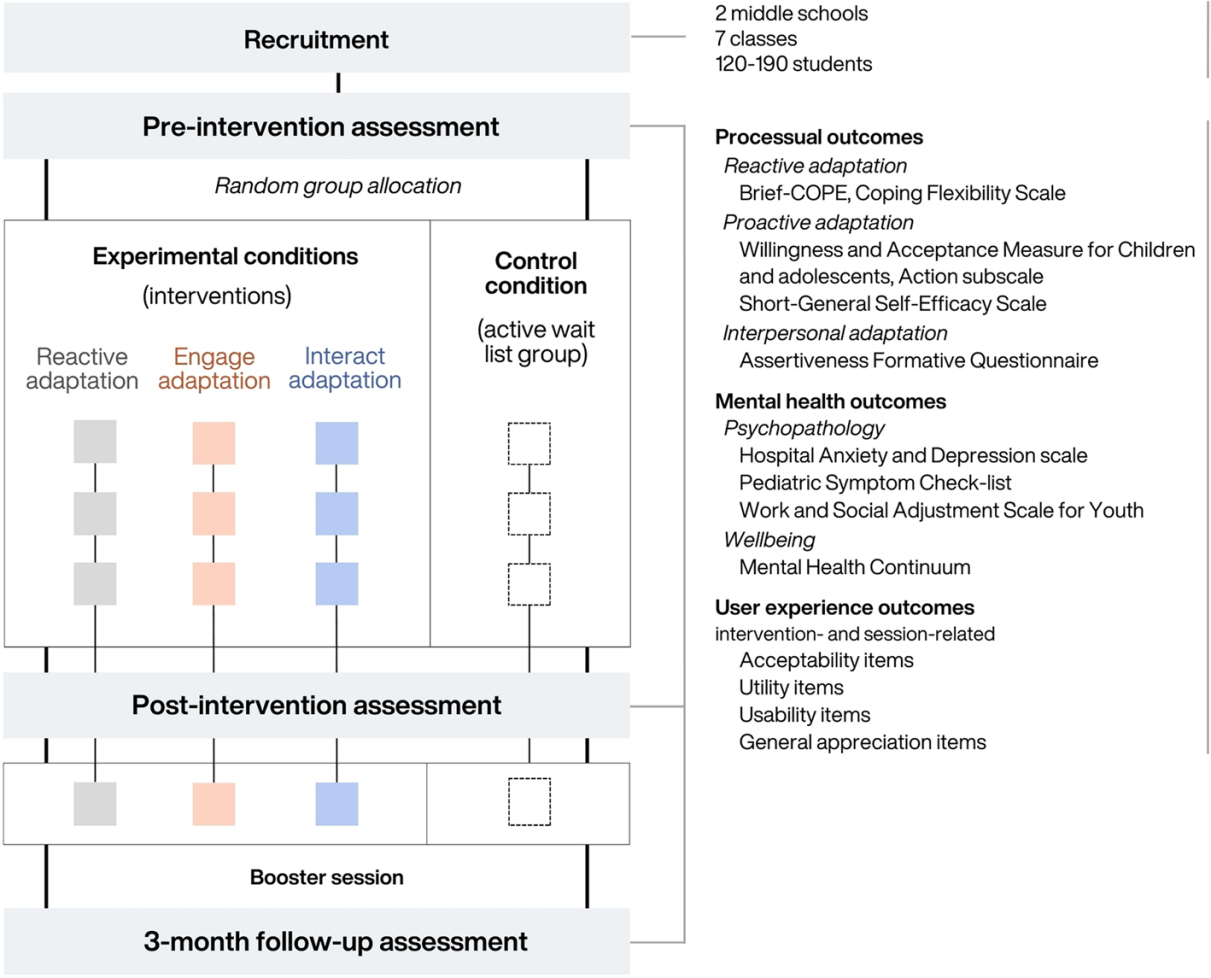
Flow diagram of main study stages

Each intervention will consist of three weekly 1-h sessions and one booster session delivered 4 weeks after the latter (see Fig. 1). Short interventions in terms of both session length and total duration are easier to implement and sustain in school settings, and booster sessions appear to enhance efficacy [34].

##### Session structure (summed up in Fig. 2)

**Fig. 2:**
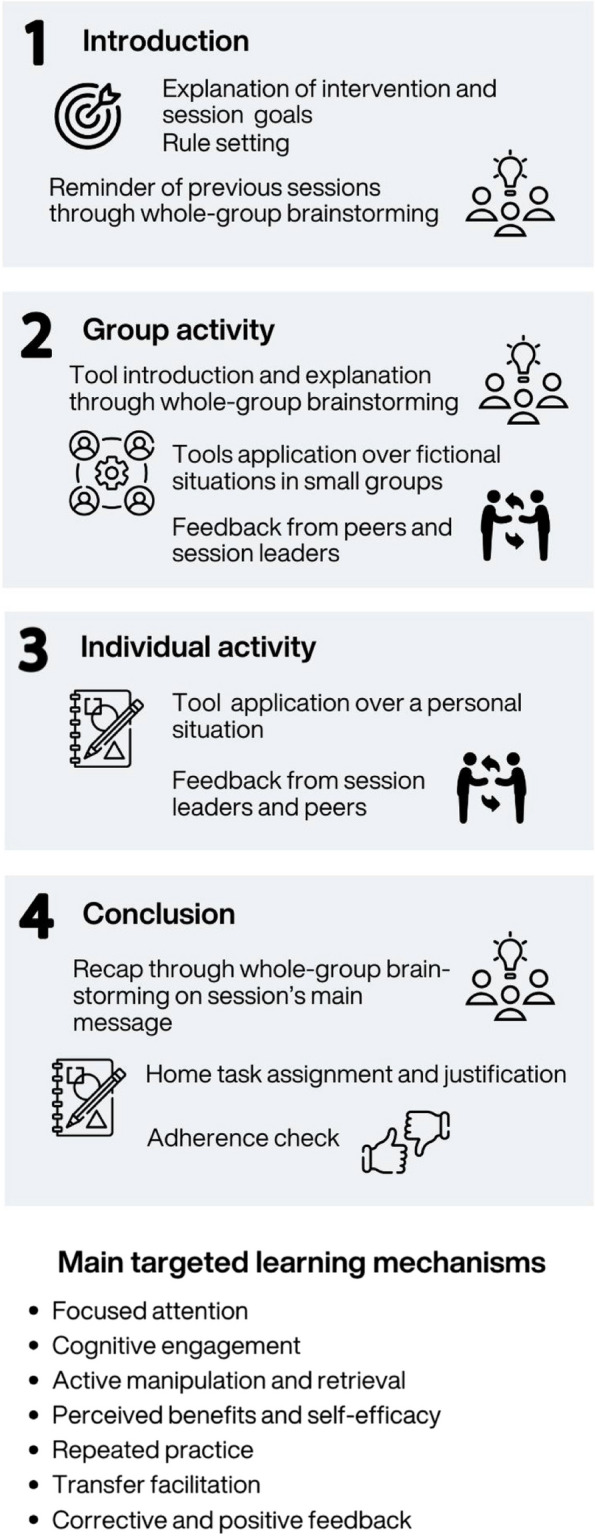
Intervention session structure and targeted cognitive learning mechanisms

A 1-h duration was chosen in the light of previous research indicating the inefficacy [203, 216] or irrelevance [223] of longer, intensive sessions, while recommending a session duration of less than 90 min to enhance efficacy [195] and acceptability [15]. Sessions in all three interventions will each follow the same sequence. They will begin with a whole-group presentation or reminder of the interventions’ goals to engage participants, and rule setting to ensure satisfactory behavior during sessions (Stage 1 in Fig. 2). Next, session content will be introduced through alternating whole-group brainstorming and small-group activities under supervision (Stage 2 in Fig. 2). Afterwards, participants will individually perform an exercise replicating previous activities, again under supervision (Stage 3 in Fig. 2). Sessions will end with brainstorming to sum up what has been learned and its daily-life usefulness, a home task assignment, and short questionnaire completion (adherence check; Stage 4 in Fig. 2).

The session structure and activities have been designed according to scientifically informed pedagogical principles (summed up at the bottom of Fig. 2). Hence, all the sessions will follow a progressive and repetitive format to allow for distributed learning [10, 111] and a graduate increase in learning complexity [59]. For pedagogical purposes, each session will recall the content previous sessions, while introducing new elements. This is also intended to reduce cognitive load [13, 163], and enhance participants’ perceived ability to successfully perform the activities [59, 128, 171], and, thus, their engagement in the learning tasks. An explicit teaching model has been adopted to promote participants’ motivated involvement, and learning transfer to real-life situations. This teaching model involves explicitly explaining to participants the objectives of the intervention and the individual sessions, as well as reasoning stages, here in the form of brainstorming and activities [32, 84, 171].

The session structure will leave plenty of place for active and interactive participation, and participants’ reflexive reasoning through group and individual brainstorming and activities, with exercises where the tools that have been learned tools can be applied to concrete situations. Active participation is assumed to foster focused attention [232] and deep information construction, manipulation [45, 137, 180], and retrieval [23, 209]). Giving participants an active learning in learning content has been identified as an important learning factor, in both educational and clinical settings (e.g., [55, 84, 186]. Large group and small group activities are also used to promote exchanges and both expert and peer modeling and exchanges [59, 84, 224]. This allows for corrective feedback and vicarious learning [164, 176, 239], in a context (i.e., adolescence) where peers exert particular influence [185]. Moreover, activities will allow students to apply the tools they have learned to their own, relevant, real-life situations, to enhance personalization and appropriation in learning [120, 143, 186]. Finally, participants will be asked to complete home assignments that reproduce in-session activities in new real-life personal situations, to further support repeated learning, transfer, and automatization [32, 84, 109].

Brainstorming and discussions between session leaders and participants will be based on Socratic questioning techniques to enhance information construction and reasoning [45, 180]. Hence, expert-guided reasoning steps will be deduced by adolescents through questioning. Interactions between session leaders and participants will be based on an empathetic, considerate, and reinforcing relational stance and on positive reinforcement from session leaders’, in order to support participants' engagement in the sessions [15, 171, 185]. Making adolescent participants feel that their opinion is considered, respected and valued, through active listening, should promote a positive attitude toward-and engagement in-session’s content and learning [235].

#### Interventions’ content (content of specific sessions provided in Table 1).

**Table 1:** Interventions content

**Sessions**	**Interventions**		
	**Reactive adaptation** ***Adapt***	**Proactive adaptation** ***Engage***	**Interpersonal adaptation** ***Interact***
1	• Functional analysis of situation, reaction, and consequences • Analysis of situation-specific locus of control	• Generation of personally valued general and specific goals • Step-by-step hierarchical planning toward goal through formulation of concrete actions	• Functional analysis of situation, reaction, and consequences, from the point of view of two protagonists
2	• Formulation of coping strategies • Assessment of strategies’ efficacy	• Identification of relevant internal and external resources • Formulation of on-moment motivation strategies • Assessment of strategies’ efficacy	• Formulation of social strategies • Assessment of strategies’ efficacy • Formulation of alternative back-up strategies
3	• Formulation of alternative back-up strategies • Discovery of new coping strategies based on positive psychology	• Identification of possible internal and external obstacles • Formulation of internal back-up strategies and alternative actions	• Formulation of new conflict resolution social strategies • Discovery of new proactive social strategies
Booster	• Reminder of whole intervention and application	• Reminder of whole intervention and application	• Reminder of whole intervention and application

##### Reactive adaptation (Adapt) intervention

*Session 1* will train participants to apply a cognitive-behavioral understanding of their reactions to common emotional situations, and their positive and negative consequences (inspired by CBT functional analysis; Psychoscop). Participants will then learn to analyze the specific locus of control in given situations (i.e., whether they have control over personal reactions, objective trigger, or both; Control Diagnosis). In *Session 2*, they will learn to look for personal strategies that might be helpful in given situations (before, during and after), depending on the locus of control, and to think about the new consequences that may arise from these strategies. Activities and aids will allow both participants to put forward creative propositions and learn about CBT-based strategies (Strataid), including defusion, cognitive restructuring, physical and emotional cooling, emotion acceptance, behavioral activation, social support seeking, and problem-solving. Finally, *Session 3* will add a critical assessment and check-up (Stratest) of renewed consequences (e.g., ‘I feel better’, ‘The situation is fixed’, etc.), leading to (non) validation of the proposed strategies. If initial strategies prove not to be effective according to their own analysis, students will be trained to look for alternate ones (Bis Strategies) and follow the previous steps until they reach Stratest. During Session 3, new strategies inspired by positive psychology principles will also be offered to students (e.g., savoring, gratitude, sources of pride, attending to positive events). Each newly learned tool will be the subject of autonomous between-sessions home assignments on new personal real-life situations. Finally, the fourth (booster) session will provide an active reminder of the whole reactive adaptation process (from Psychoscop to Bis Strategies).

##### Proactive adaptation (Engage) intervention

In *Session 1*, participants will first be trained to identify and link general, personally relevant and valued goals (Macrobjectives) to important psychological needs (e.g., autonomy, self-esteem, positive relationships, purpose, positive emotions) and to happiness. Second, they will learn to split their general goal into more specific ones (Microbjectives), and then identify concrete, progressive and realistic actions that will allow them to attain each one (Steps to Take). *Session 2* will be dedicated to the identification of participants’ personal internal (e.g., strengths, skills) and external (e.g., persons, places, services) resources that could help them to attain their specific goals. Participants will also be trained to identify cognitive (e.g., self-talk, visualization) and behavioral (e.g., preparation, training) strategies that could help them to effectively carry out previously defined actions. In *Session 3*, students will learn to first identify possible internal (e.g., given thoughts and emotions) and external (e.g., unexpected events) obstacles (Red Lights) that could prevent them from effectively carrying out previously defined actions. They will then learn to look for strategies (e.g., positive thoughts, alternative emotions and actions) to help them bypass these obstacles (Green Lights, Bis Itinerary). Finally, they will be led to critically analyze their strategies’ efficacy, and appraise their efforts with a positive gloss (e.g., ‘What have I done so far? Which objectives have I attained? Which benefits have I derived from them?’). Home assignments will allow participants to apply their newly learned tools to new personal goals between each session. Finally, the fourth (booster) session will provide an active reminder of the whole proactive adaptation process (from Macrobjective to Bis Itinerary).

##### Interpersonal adaptation (Interact) intervention

In *Session 1*, students will be trained to understand others’ reaction, and explain it in terms of links between social triggers, thoughts, emotions, and behavior. Participants will then be trained to recognize interrelations between protagonists’ reactions (*coupled* functional analyses; Mirrored-Psychoscop), and to critically assess the positive and negative consequences of protagonists’ reactions (for themselves, for the other, and for the relationship). In *Session 2*, adolescents will first learn to look for strategies (before, during and/or after the situation) that may help them to reduce the negative consequences and increase the positive ones in a given social situation. Students will have access to strategies inspired by self-assertiveness CBT techniques (Strataid; e.g., *I* speaking, expressing feelings and needs, asking, apologizing, refusing, inquiry, prosocial actions, looking for a mediator, attending to nonverbal cues). They will also be able to suggest strategies of their own, providing they maintain a *relational balance* between the two protagonists, to avoid passive or aggressive deviations. Second, participants will be trained to think of new consequences arising from these alternative reactions and to assess the effectiveness of their (Stratest; e.g., ‘The situation is fixed’, ‘I feel better’, ‘The other feels better’). They will also practice identifying alternative strategies if the first ones seem ineffectual (Bis Strategies). *Session 3* will be devoted to repeating previous procedures with a focus on situations of outright conflict, adding specific conflict-solving strategies to Strataid. This session will end with the identification of “positive strategies” aimed at proactively enhancing relationships through spontaneous prosocial behaviors (i.e., apart from any triggering event). These positive strategies may include expressing gratitude, looking for positive qualities in others, complimenting, proposing social activities, performing acts of kindness, and displaying positive nonverbal cues. Finally, the fourth (booster) session will allow an active recall of the whole social adaptation process (from Mirrored-Psychoscop to Bis Strategis and conflict resolution strategies).

#### Intervention material

To introduce, work on, and train adaptive processes, various situations will be used as example to enhance the intervention’s perceived utility to participants (favoring motivated engagement in activities). This should also support learning transfer in diverse contexts [8, 59, 84]. Varied and realistic [72, 143] examples of situations have been chosen for use in sessions for their developmental and emotional (but not distressing) relevance to adolescents’ usual concerns [122], addressing challenges they may encounter in the real world [236]. They include, for example, losing in sports matches or video games, having a math test, being late for the first class, parents’ refusal to let them go on an outing, or a friend sitting next to someone else.

Aids are intended to 1) cognitively engage participants in the activities, and 2) provide relevant information related to session content. Moreover, media have been designed specifically to ensure simplicity and clarity for adolescents [32, 84, 236], in order to enhance understandability and reduce cognitive load [13, 163]*.* Materials feature a multimedia component (i.e., written and oral verbal format combined with picture format), to enhance both cognitive learning and learning motivation [7, 138, 139]. Attractiveness and ease of use have been emphasized, to foster participants’ engagement in activities [143, 185, 200].

In addition to the interactive in-session materials, participants will be given intervention workbooks (inspired by the diary format). These workbooks will contain key information (session reminders, additional information), as well as exercises they will have to complete both during and between sessions (home tasks), as a way of supporting repeated learning, practice and transfer [59, 84, 209]. Participants will also have access to a dedicated website providing further resources related to the content of their allocated intervention (e.g., additional exercises), as well as supplementary general thematic resources (e.g., guided relaxation and meditation audio recordings).

Intervention content and materials can be supplied (in French) free of charge, on request to the corresponding author, under the terms of a CC-BY-NC-SA license (implying the crediting of authors, identification of any additions or modifications, nonprofit use, and sharing of materials under the same CC license conditions). A sample of in-session media can be consulted in Supplementary Material (2).

### Study design

A four-arm randomized controlled trial will be conducted during the 2023–2024 school year (see Fig. 2). Seven fourth-grade classes from two French urban schools will be involved in the research process, and will constitute of randomization. Intervention allocation will be carried out in such a way as to ensure that each school benefits from all three interventions. Study information will be delivered to students and parents by one of the investigators through oral presentations (class sessions, back-to-school meetings) and documentation. Written informed consent will be collected using either printed forms (students and parents) or online forms (parents only, depending on their preference), giving respondents sufficient time to ask investigators for details or explanations (face-to-face or via email). Even if students and/or parents (legal guardians) do not give their assent/consent, students will still benefit from the intervention; however, in this case, they will not complete questionnaires.

Questionnaire completion should take about 20–30 min for students, and will take place in the students’ school during school time. Parents (or legal guardians) will answer questionnaires either on printed forms distributed during meetings, to be returned to the school, or on online forms accessible via a QR code (depending on their preference). These will take about 10 min. Above and beyond their conceptual and psychometric relevance, the measures described above have been chosen for their relative shortness, to reduce effort and fatigue for participants during completion. They will be submitted to participants before the intervention (pre-intervention assessment), after the third session (post-intervention assessment), and 3 months later (follow-up assessment).

Each class will be divided into two equal pools of students, based on the alphabetic list of students (after removal of students whose assent/consent will have not been given or collected, and before any data analysis). The first half of the list will be randomly attributed (with use of computer-generated random numbers) to one of the three experimental conditions (i.e., Reactive, Proactive, or Interpersonal adaptation intervention), ending up with at least two classes per intervention (one per school), while the second half will constitute the control group (wait list). For all intervention groups, sessions will begin 1 week after the pre-intervention assessment. The three 1-h sessions will be conducted over 3 consecutive weeks, followed by the post-intervention assessment the week after, and a booster session 1 month later (with the follow-up assessment 2 months after booster session). Sessions will take place in classrooms during school time (e.g., form time) during the first semester of the school year. Interventions will be delivered to half-class groups of students (about 15 participants per group). The intervention groups (see below) will be overseen by a clinical psychologist and PhD student (main leader; one of the investigator) and a Master student in clinical psychology (co-leader), both CBT-trained, as session leaders specializing in mental health have been shown to improve the efficacy of school-based interventions [224, 225]. Active control group sessions will be led by two psychology undergraduates and will feature activities unrelated to mental health (see below). Control group participants will be offered serious boardgames intended to train purely cognitive abilities such as attention, categorization, logical reasoning, working memory, and cognitive flexibility. The sessions’ format will be identical to that of the intervention sessions, in terms of duration, frequency and number (three weekly 1-h sessions plus one booster session), number of participants, and use of both group and individual activities. For ethical reasons, the control group will benefit from the same intervention as the other half of their class after the end of the study, with no data collection, in the last part of the school year (i.e., after follow-up assessments).

### Data analysis

Mixed linear models (or nonparametric equivalent tests, depending on data normality and homogeneity in the sample) will be calculated using R^©^ software to test the Time x Intervention effects, with a 5% significance threshold. The analysis will be run while controlling for school, sociodemographic data (e.g., gender, socioeconomic status, current and previous difficulties and treatments), and participants’ in-session engagement.

## Discussion

To our knowledge, this will be the first study to separately assess three original, universal, school-based prophylactic interventions, based on scientific theories (CBT) and user needs, conducted among middle-school students in France. As such, it may help to fill several gaps. First, by demonstrating the effectiveness of the interventions and providing free access to their full content, this study to encourage the implementation of prophylactic interventions on students’ mental health in France, where more efforts are still needed [125], specifically for middle-schoolers [104, 126]. In addition, the transversal content, coupled with the short duration of the interventions, could facilitate large-scale dissemination in settings with limited resources [71]. Second, results will provide new insights into the roles of various processes in adolescent psychopathology and wellbeing, which may help to enhance the efficacy of the interventions that are currently available [131, 160]. Depending on results, further studies may be able to validate or adapt these interventions delivered to specific populations (e.g., rural schools, populations with diverse economic and cultural backgrounds, and older and younger adolescents). In conclusion, this study will pursue research and efforts to apply results, in order to provide schools with more parsimonious, efficient, ecological, and user-relevant prophylactic interventions.

## Supplementary Information

**Additional file 1. **Items related to the various dimensions of the user experience questionnaire, depending on assessment timepoint and type of respondent.

**Additional file 2. **Aids provided to participants for each intervention.

## Data Availability

This research is currently being conducted and data collection is in progress. Assessment tools and intervention materials can be provided in French for free on request to the corresponding author.
