# Supplementary material for: Effects of three prophylactic interventions on French middle-schoolers’ mental health: protocol for a randomized controlled trial
Source: BMC Psychol. 2024 Apr 13;12:204. doi: 10.1186/s40359-024-01723-8 (PMC11016224; doi:10.1186/s40359-024-01723-8)
Supplement: Supplementary file 1 — Additional file 1. Items related to the various dimensions of the user experience questionnaire, depending on assessment timepoint and type of respondent. [file 40359_2024_1723_MOESM1_ESM.zip › Supplementary_Material_1.Table1..pdf]

Table 1.

Items from user experience questionnaires for the whole intervention, according to dimension.

	Pre-test	Post-test	Follow-up
	**ACCEPTABILITY**		
**Students**	Is part of the school's missions<br>Is worth devoting school time	*Same as pre-test +*<br>Sessions too long/too short<br>Ideal session duration [ ]<br>Too much/not enough sessions<br>Ideal number of sessions [ ]	*Same as pre-test*
**Parents**	Is part of the school's missions<br>Is worth devoting school time<br>Think it would be good that workshops this kind for parents would be proposed at school after school/on Saturday morning, for [ ] sessions lasting [ ] hours		/
**Head teacher**	Is part of the school's missions<br>Is worth devoting school time<br>Would be interested in animating this kind of interventions<br>As long as I have everything ready to go<br>As long as I am trained by a mental health professional<br>As long as I am supervised by a mental health professional<br>As long as it doesn't take more than [ ] hours of training,<br>[ ] hours of animation, [ ] hours of supervision		/
	**UTILITY**		
	**Pre-test**	**Post-test**	**Follow-up**
**Students**	Useful<br>Necessary	*Same as pre-test +*<br>The workshops have helped me<br>Have learned things<br>Have used / been willing to use what we learnt in my daily life<br>Am willing to use what we learnt in my daily life when i will need it later on<br>Have used the workbook<br>Have used the website<br>The workbook have helped me<br>The website was useful	In hindsight, workshops have helped me<br>In hindsight, have learned things<br>In hindsight, what we learnt can be useful to me in daily life<br>Have been willing to use what we learnt in my daily life sometimes<br>Have reused the workbook<br>Have reused the website<br>Am willing to use what we learnt in my daily life when i will need it later on
**Parents**	Useful<br>Necessary		/
**Head teacher**	Useful<br>Necessary		/
	**USABILITY**		

	Pre-test	Post-test	Follow-up
**Students**	/	Have understood what we have learned in the workshops<br>Have been able to use what we learnt in my daily life<br>Feel able to use what we learnt in my daily life when I will need it later on<br>Found easy to understand and use the aids<br>Found easy to understand and to do the activities<br>Found the workbook easy to use	Have better understood some things with time<br>Have been able to use what we learnt in my daily life sometimes<br>Feel able to use what we learnt when I will need it
**Parents**	/	/	/
**Head teacher**	/	/	/
	**GENERAL APPRECIATION**		
	**Pre-test**	**Post-test**	**Follow-up**
**Students**	Glad to participate<br>Interested<br>Curious	Glad to have participated<br>Interested in the workshops<br>Positive effects due to the workshops [ ]<br>Negative effects due to the workshops [ ]<br>Enjoyed the aids<br>Enjoyes the activities<br>Think it would be good for other students to do those workshops<br>Would like to do these workshops again later<br>Would like to do the same kind of workshops on other topics [ ]<br>Overall rating [ ]/10	Glad to have participated<br>In hindsights, positive effects due to the workshops [ ]<br>In hindisghts, negative effects due to the workshops [ ]<br>Think it would be good for other students to do those workshops<br>Would like to do these workshops again later<br>Would like to do the same kind of workshops on other topics [ ]<br>Overall rating [ ]/10
**Parents**	Glad that my child participates<br>Interested<br>Curious<br>Think it would be good if workshops this kind could to be repeated during the year/from one year to the other<br>Think it would be good for students to do workshops this type on other subjects such as [ ]	Glad that my child participated<br>Think it would be good if workshops this kind could to be repeated during the year/from one year to the other<br>Think it would be good for students to do workshops this type on other subjects such as [ ]	/
**Head teacher**	Glad that my class participates<br>Think it would be good if workshops this kind could to be repeated during the year/from one year to the other<br>Think it would be good for students to do workshops this type on other subjects such as [ ]	Glad that my class participated<br>Think it would be good if workshops this kind could to be repeated during the year/from one year to the other<br>Think it would be good for students to do workshops this type on other subjects such as [ ]	/
**Total number of items**			
**Students**	7	36	19
**Parents**	10	8	/

**Head teacher**	12	12	/

Note. The / symbol indicates that the dimension will not be evaluated. The [ ] symbol indicates that more details were asked to respondents. Respondents will rate their level of agreement with items on a 5-point Likert scale from 0 ('Not true at all') to 4 ('Absolutely true').
For concision concern, items' formulation is not exactly those which will be submitted to participants. Full questionnaires can be provided in French on request to investigators.
