# Supplementary material for: Effects of three prophylactic interventions on French middle-schoolers’ mental health: protocol for a randomized controlled trial
Source: BMC Psychol. 2024 Apr 13;12:204. doi: 10.1186/s40359-024-01723-8 (PMC11016224; doi:10.1186/s40359-024-01723-8)
Supplement: Supplementary file 1 — Additional file 1. Items related to the various dimensions of the user experience questionnaire, depending on assessment timepoint and type of respondent. [file 40359_2024_1723_MOESM1_ESM.zip › Supplementary_Material_1.Table2..pdf]

Table 2.

Items from user experience questionnaires for specific sessions, according to dimension (adherence check administered to students at end of each session).

**GENERAL APPRECIATION**
Have really disliked / Have really liked
**UTILITY**
Not useful at all / Very useful
Have learned nothing at all / Have learned a lot of things
Not willing at all / Very willing to use what I have learned in daily life
**UTILISABILITY**
Haven't understood anything / Have understood everything
**COGNITIVE ENGAGEMENT**
Very bored / Very interested
Very distracted / Very focused
Not active at all / Very active
**OTHER**
Did the home task since the last session

Note. Respondents will rate their level of agreement with items on a 4-point Likert scale from 0 (« proposition ») to 4 (« opposite proposition »).
For concision concern, items' formulation is not exactly those which will be submitted to participants.
Full questionnaires can be provided in French on request to investigators.
