# Supplementary material for: Effects of three prophylactic interventions on French middle-schoolers’ mental health: protocol for a randomized controlled trial
Source: BMC Psychol. 2024 Apr 13;12:204. doi: 10.1186/s40359-024-01723-8 (PMC11016224; doi:10.1186/s40359-024-01723-8)
Supplement: Supplementary file 2 — Additional file 2. Aids provided to participants for each intervention. [file 40359_2024_1723_MOESM2_ESM.zip › Supplementary-Material_2.1.Aids_ADAPT.pdf]

PSYCHOSCOPE

SITUATION

les faits objectifs

ÉMOTIONS & SENSATIONS

dans cette situation,
je me sens...

PENSÉES

dans cette situation,
je me dis que...

ACTIONS

dans cette situation,
ma réaction est de...

PSYCHOSCOPE

CONSÉQUENCES POSITIVES (+)

pour soi • pour les autres • pour la relation

CONSÉQUENCES NÉGATIVES (−)

CONSÉQUENCES POSITIVES BIS (+)

pour soi • pour les autres • pour la relation

CONSÉQUENCES NÉGATIVES BIS (−)

STRATÉGIES

	Avant	Pendant	Après
1.	☐	☐	☐
2.	☐	☐	☐
3.	☐	☐	☐
4.	☐	☐	☐
5.	☐	☐	☐
6.	☐	☐	☐

STRATEST

- ☐ Je me sens mieux ____________________
- ☐ Le problème est résolu ____________________
- ☐ La situation est améliorée ____________________
- ☐ J'ai évité des ennuis, des soucis ____________________
- ☐ J'ai gagné quelque chose ____________________
- ☐ Je suis content-e de moi ____________________
- ☐ Autre (préciser) ____________________

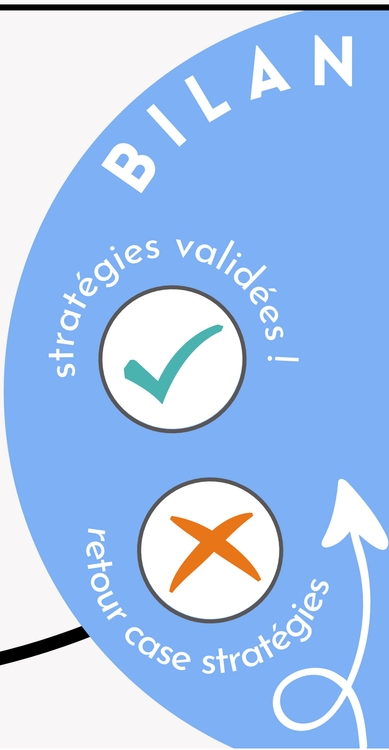

STRATAIDE

Nota Bene : Je peux **combiner autant de stratégies** que je le veux !

① Je veux **me sentir mieux, diminuer l'impact de mes pensées ou émotions**

par les pensées

DÉFUSIONNER

Les émotions et les pensées ne commandent pas mes actions

Ce n'est pas parce que je pense ou ressens ça que c'est vrai

ACCEPTER

C'est OK d'avoir des émotions ou pensées négatives, elles sont normales et pas dangereuses

D'autres peuvent penser ou ressentir des choses similaires

PENSER À AUTRE CHOSE

Recette de cuisine
Compter à l'envers
Faire des listes
Mes 5 sens
Un souvenir positif

à haute voix ou dans ma tête

PENSER DIFFÉREMMENT

Une pensée qui m'aiderait : ...
utile, rassurante, réaliste

Ce n'est pas grave, parce que...

C'est aussi positif, parce que...

Je vais gérer la situation, car...

Si ça arrivait à un-e ami-e, je lui dirais...

par les actions

ME DISTRAIRE

Une activité
qui me fait du bien
seul-e ou **avec des proches**

laquelle ? avec qui ?

M'APAISER

STOP
Temps de pause
Observer
Poursuivre

Respirer lentement (5-5)

Un exercice de relaxation

DÉCHARGER

Faire une **activité physique** *laquelle ?*

Crier/frapper dans un oreiller

Contracter mes muscles pendant 10s

Utiliser un **antistress** *quoi ?*

② Je veux **améliorer la situation**

modifier les conséquences

éviter qu'elle ne se répète

MON OBJECTIF

Ce que je voudrais pour la situation

TOUTES LES SOLUTIONS POSSIBLES

faire une liste ~ avec créativité !

CHOISIR

La ou les meilleures solutions

PAS SATISFAIT-E ?

J'essaie autre chose...
ou je trouve d'autres solutions !

UTILISER L'AIDE DES AUTRES

Quelqu'un qui peut m'aider à résoudre la situation

Quelqu'un de confiance pour m'écouter

qui ?

STRATÉGIES BIS

	Avant	Pendant	Après
1b.	☐	☐	☐
2b.	☐	☐	☐
3b.	☐	☐	☐
4b.	☐	☐	☐
5b.	☐	☐	☐
6b.	☐	☐	☐

STRATAIDE +

A LA RECHERCHE DU POSITIF

Qu'est-ce qui va se passer de bien...

- aujourd'hui ?
- cette semaine ?

Qu'est-ce qui s'est passé de bien...

- aujourd'hui ?
- cette semaine ?

LES PÉPITES DU JOUR

Lister 3 choses, moments, sensations
que j'ai vraiment appréciés
à savourer de nouveau

LES FIERTÉS

De quoi je peux être fier/fière...

- aujourd'hui ?
- cette semaine ?

MERCI...

Lister :

- **3 choses dont je suis reconnaissant-e**
  (à quelqu'un, la vie...)
- **3 personnes que je voudrais remercier**
  pour ce qu'elles m'apportent ou m'ont donné

STRATÉGIES BIS

Avant Pendant Après

	Avant	Pendant	Après
1b.	☐	☐	☐
2b.	☐	☐	☐
3b.	☐	☐	☐
4b.	☐	☐	☐
5b.	☐	☐	☐
6b.	☐	☐	☐

STRATAIDE +

A LA RECHERCHE DU POSITIF

Qu'est-ce qui <u>va se passer</u> de bien...

- aujourd'hui ?
- cette semaine ?

Qu'est-ce qui <u>s'est passé</u> de bien...

- aujourd'hui ?
- cette semaine ?

LES PÉPITES DU JOUR

Lister 3 choses, moments, sensations que j'ai vraiment appréciés

à savourer de nouveau

MERCI...

Lister :

- **3 choses dont je suis reconnaissant-e**
  (à quelqu'un, la vie...)
- **3 personnes que je voudrais remercier**
  pour ce qu'elles m'apportent ou m'ont donné

LES FIERTÉS

De quoi je peux être fier/fière...

- aujourd'hui ?
- cette semaine ?
