# Supplementary material for: Effects of three prophylactic interventions on French middle-schoolers’ mental health: protocol for a randomized controlled trial
Source: BMC Psychol. 2024 Apr 13;12:204. doi: 10.1186/s40359-024-01723-8 (PMC11016224; doi:10.1186/s40359-024-01723-8)
Supplement: Supplementary file 2 — Additional file 2. Aids provided to participants for each intervention. [file 40359_2024_1723_MOESM2_ESM.zip › Supplementary-Material_2.2.Aids_ENGAGE.pdf]

ADOBJECTIFS

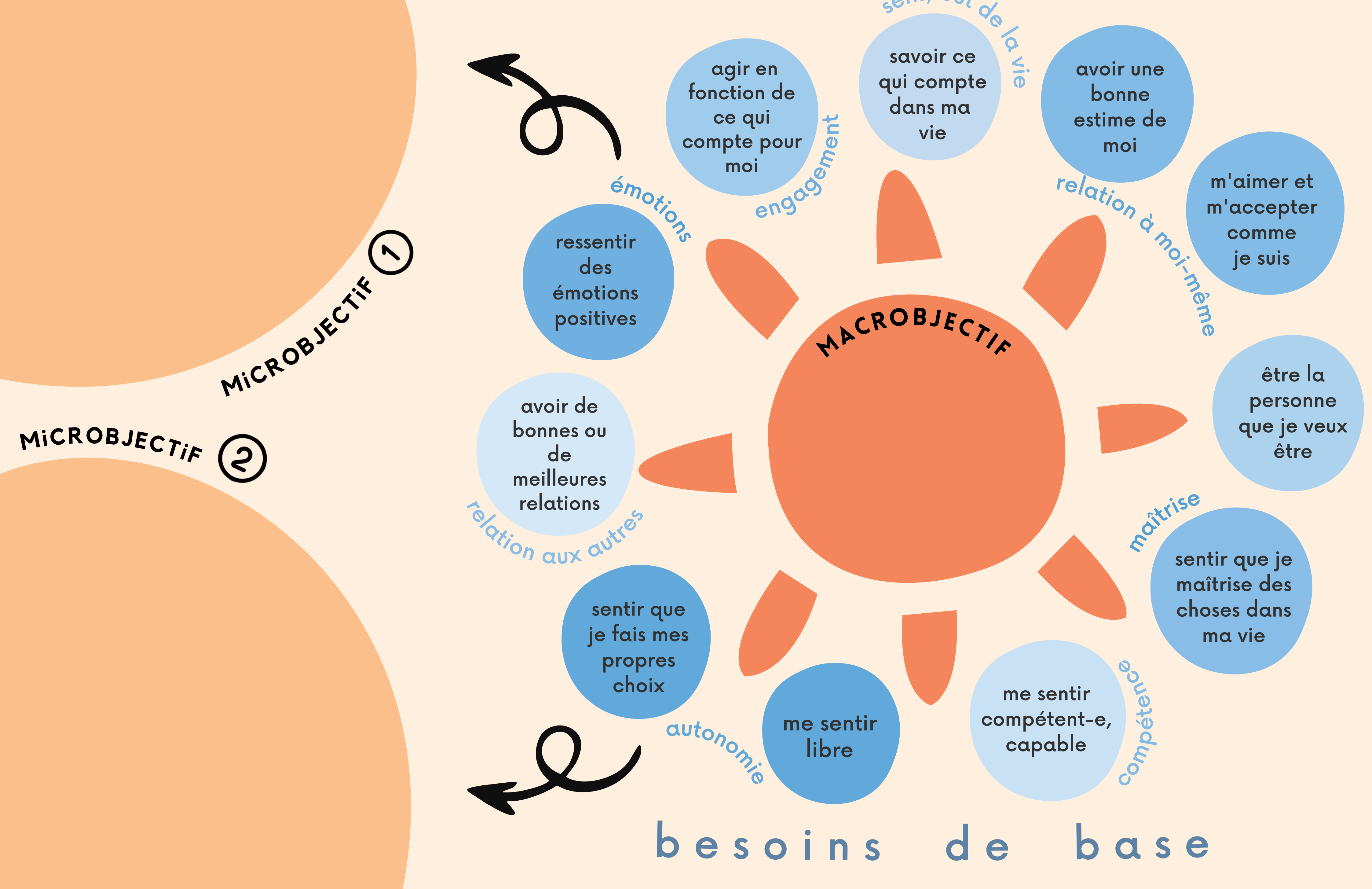

STRATEST

−	+

- ☐ J'ai atteint l'objectif
- ☐ Je me suis rapproché de l'objectif
- ☐ J'ai développé des forces
- ☐ Je me suis entraîné-e, préparé-e
- ☐ J'ai répondu à un/des besoins
- ☐ Autre (préciser)

BILAN

stratégies validées!

retour cases Marche à suivre

CHERCHER

BILAN +

Comment j'ai **progressé**

Ce que j'ai **déjà accompli**

Les **bénéfices** je tire du changement

STRATAIDE

par des pensées

ME PRÉPARER
M'imaginer en train de réaliser / réussir l'action

M'ENCOURAGER
en me disant que… car…

ME RASSURER
en me disant que… car…

ME RAPPELER POURQUOI
ça en **vaut la peine**/c'est **important**, car…
ça va **me permettre** de…

ME FÉLICITER
en me disant que… car…

ÊTRE FIER-ÈRE
d'avoir **essayé**, **fait un pas** vers l'objectif, car…

ME RAPPELER QUE
ça en valait la peine, car…
c'est normal de ne pas réussir à chaque fois, car…

par des actions

ME DÉTENDRE
respirer, **me relaxer**, **me concentrer**, méditer

CHERCHER DE L'AIDE
des personnes, des ressources

M'ENTRAÎNER
répéter l'action auparavant, demander des **retours** à quelqu'un

ME PRÉPARER
physiquement, en rassemblant ce dont j'ai besoin

JOKER : REFORMULER L'OBJECTIF

stratégies combinables !

MARCHE À SUIVRE

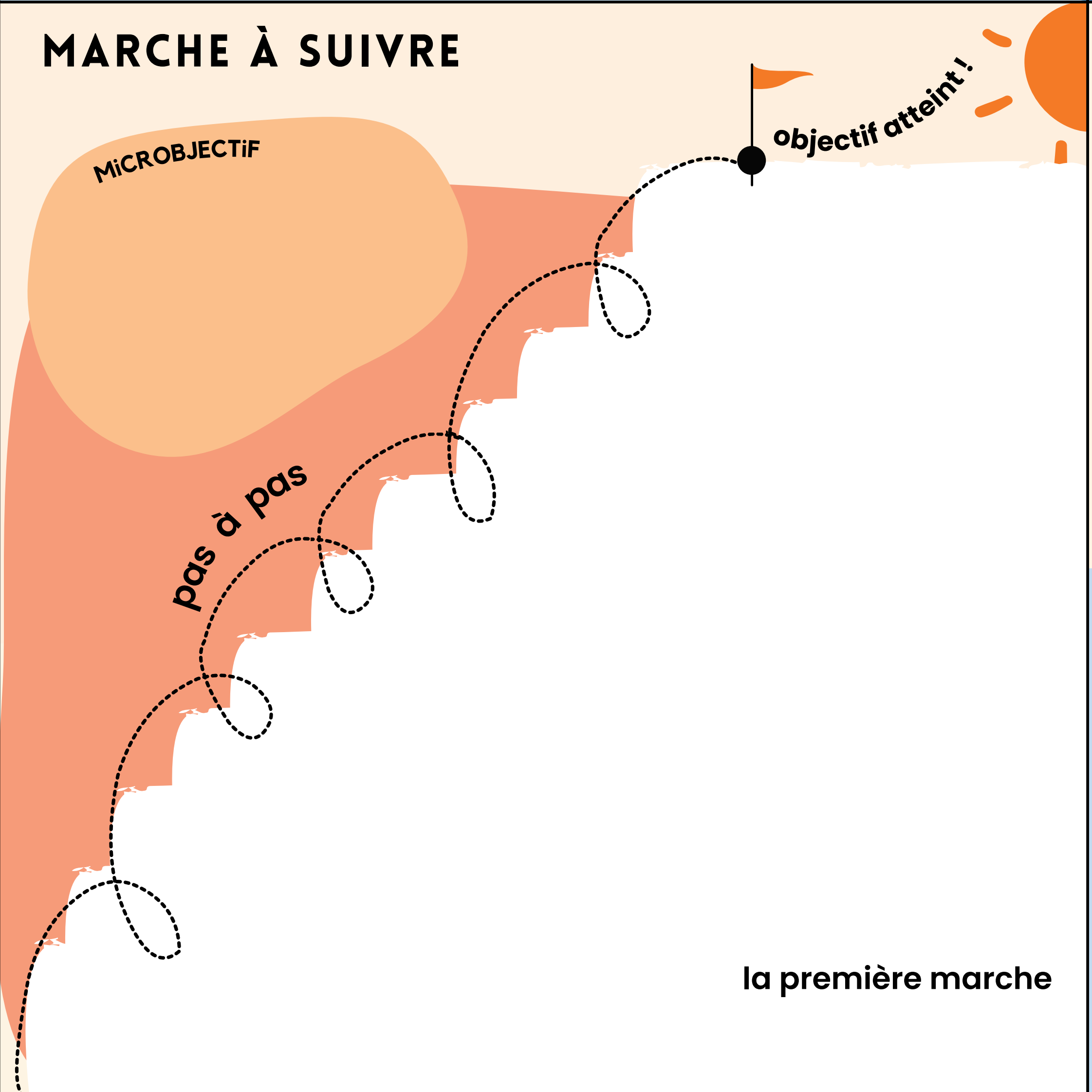

FORCES INTERNES

qualités, motivation, talents…

✓ **que j'ai déjà** » **à développer**

RESSOURCES EXTERNES

lieux, objets, services, personnes…

✓ **que j'ai déjà** » **à développer**

EXEMPLES

Quand je me dis...

"j'en suis pas capable"
"j'y arriverai jamais"
"ça sert à rien"
"je suis nul-le"
"de toute façon..."
"c'est trop dur"
"j'essaierai plus tard"
"on risque de me juger"
...

Quand je...

procrastine
m'emporte
suis distrait-e par autre chose
fais autre chose à la place

Quand je me sens...

anxieux/se, stressé-e
en colère, agacé-e
impuissant-e
...

pensées et émotions feux-rouges

mes feux-verts

EXEMPLES

- **des pensées + aidantes** *lesquelles ?*
- **des actions + utiles** *lesquelles ?*
- **des émotions + positives** *comment ?*

espoir
motivation
optimisme
confiance
énergie
calme

AVANT la marche

APRÈS la marche

STRATÉGIES

PENDANT la marche

ITINÉRAIRE BIS

obstacles possibles
