# Supplementary material for: Effects of three prophylactic interventions on French middle-schoolers’ mental health: protocol for a randomized controlled trial
Source: BMC Psychol. 2024 Apr 13;12:204. doi: 10.1186/s40359-024-01723-8 (PMC11016224; doi:10.1186/s40359-024-01723-8)
Supplement: Supplementary file 2 — Additional file 2. Aids provided to participants for each intervention. [file 40359_2024_1723_MOESM2_ESM.zip › Supplementary-Material_2.3.Aids_INTERACT.pdf]

PSYCHOSCOPE MIROIR

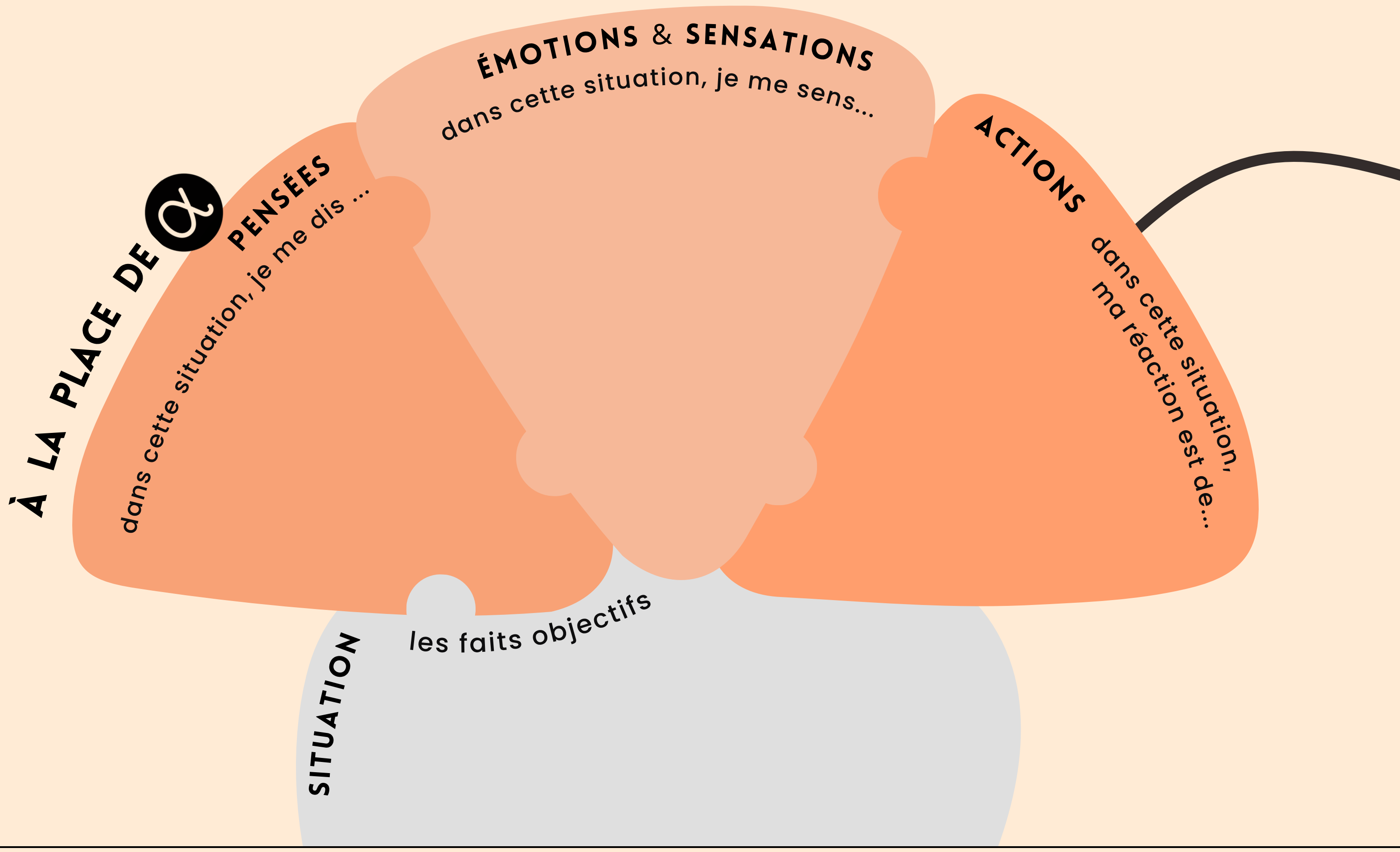

PSYCHOSCOPE MIROIR α

CONSÉQUENCES POSITIVES +

pour la relation • pour les autres • pour soi

− CONSÉQUENCES NÉGATIVES

CONSÉQUENCES POSITIVES BIS +

pour la relation • pour les autres • pour soi

− CONSÉQUENCES NÉGATIVES BIS

STRATÉGIES α

	Avant	Pendant	Après
1.	☐	☐	☐
2.	☐	☐	☐
3.	☐	☐	☐
4.	☐	☐	☐
5.	☐	☐	☐
6.	☐	☐	☐

STRATEST α

- ☐ est satisfait-e
- ☐ est satisfait-e
- ☐ et s'entendent mieux
- ☐ Le problème est résolu
- ☐ La situation est apaisée
- ☐ Autre (préciser)

BILAN

stratégies validées !

retour case stratégies

① STRATAIDE

Nota Bene : Je peux **combiner autant de stratégies** que je le veux !

PARLER EN JE
Je pense, **j'**ai l'impression,
je me sens...

DIRE MES ÉMOTIONS, BESOINS
Je ressens/ me sens... car...
J'ai **besoin** de... / que...

NON-VERBAL
Utiliser le ton de la voix, les gestes

FAIRE UNE DEMANDE
en décrivant **précisément**
l'action que j'attends

DEMANDER PARDON
Je suis désolé-e de...

ENQUÊTE
J'aimerais mieux comprendre...
Est-ce que **tu peux m'expliquer**...?
(**Quand**) est-ce que **tu serais disponible** pour parler de... ?

EMPATHIE
Je comprends que...
Je suis désolée que tu...

PROPOSER
Je te propose que...
Que penses-tu que... ?

REMERCIER
Merci d'avoir écouté, d'avoir accepté de faire un effort

RÉAGIR DIFFÉREMMENT
quoi ? comment ?

FAIRE UN PAS VERS L'AUTRE
Une **action** pour
se rapprocher de l'autre

AIDER L'AUTRE
Une **action** pour **rendre service**, se racheter,
réparer une faute ou une erreur

SE CALMER
S'éloigner, s'isoler, respirer lentement

INDISPONIBILITÉ
Je ne me sens pas disponible pour en parler maintenant

MÉDIATEUR
Demander l'avis ou l'intervention de quelqu'un

---

② STRATAIDE

SPÉCIAL CONFLITS

CAUSE DU PROBLÈME
- **selon** α
- **selon** Ω

LISTE DES SOLUTIONS POSSIBLES
- **pour** α
- **pour** Ω

de la meilleure à la moins bonne

EFFORTS
- **que** α
- **et** Ω

sont prêt-e à faire

PREMIER PAS
- **que** α
- **et** Ω

sont prêt-e-s à faire l'un-e vers l'autre

COMPROMIS FINAL
la meilleure solution,
à la fois pour α **et pour** Ω**,**
inspirée des idées de chacun

STRATEST

STRATÉGIES BIS α

	Avant	Pendant	Après
1b.	☐	☐	☐
2b.	☐	☐	☐
3b.	☐	☐	☐
4b.	☐	☐	☐
5b.	☐	☐	☐
6b.	☐	☐	☐

STRATAIDE +

A LA RECHERCHE DU POSITIF

Qu'est-ce que j'aime bien chez l'autre ?

- comme qualités
- comme actions

lesquelles ?

PROPOSER

une sortie,
une activité...
à partager

quoi ? à qui ?

ACT KIND

Proposer mon aide

Trouver un acte de gentillesse

quoi ?
comment ? à qui ?

FAIRE UN COMPLIMENT

Dire à quelqu'un ce que j'aime chez lui/elle (être précis-e)

une action, une réussite, son apparence...

qui ? quoi ?

UTILISER MON CORPS

avec un sourire,
une expression,
ma voix,
un geste...

MERCI

Remercier l'autre pour quelque chose qu'iel a dit ou fait,
qui m'a aidé-e, touché-e...

qui ? pour quoi ?

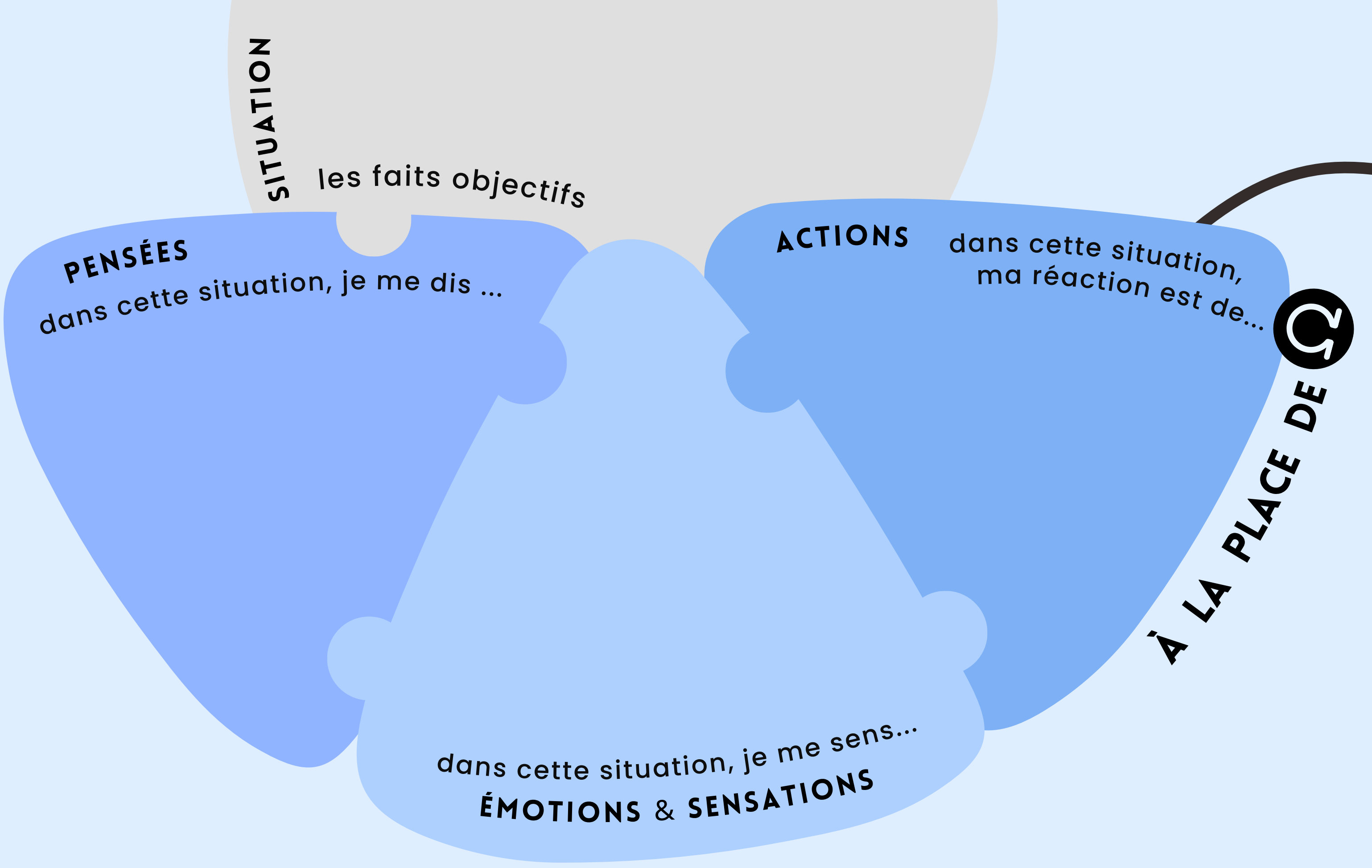

PSYCHOSCOPE MIROIR

PSYCHOSCOPE MIROIR Ω

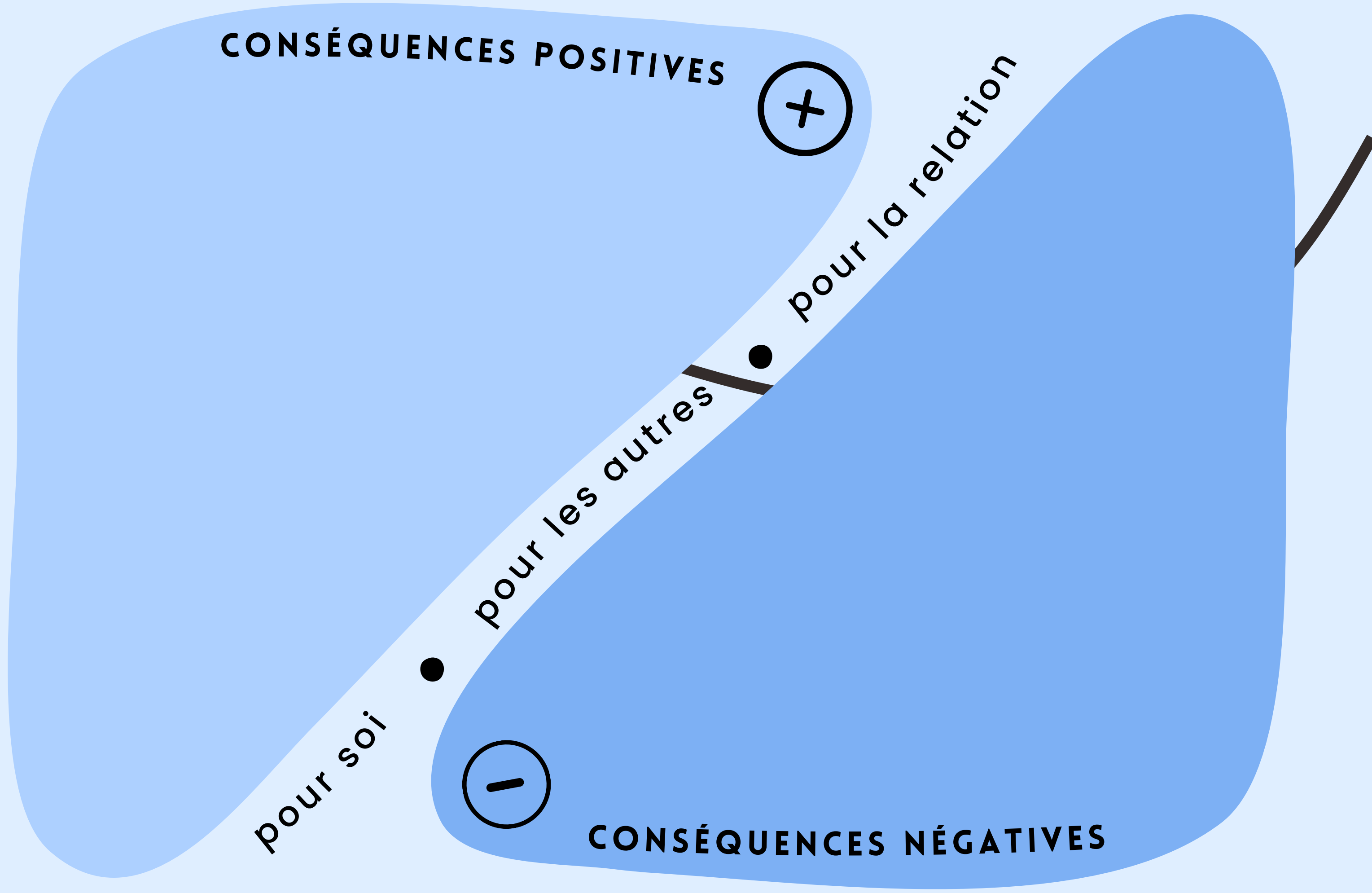

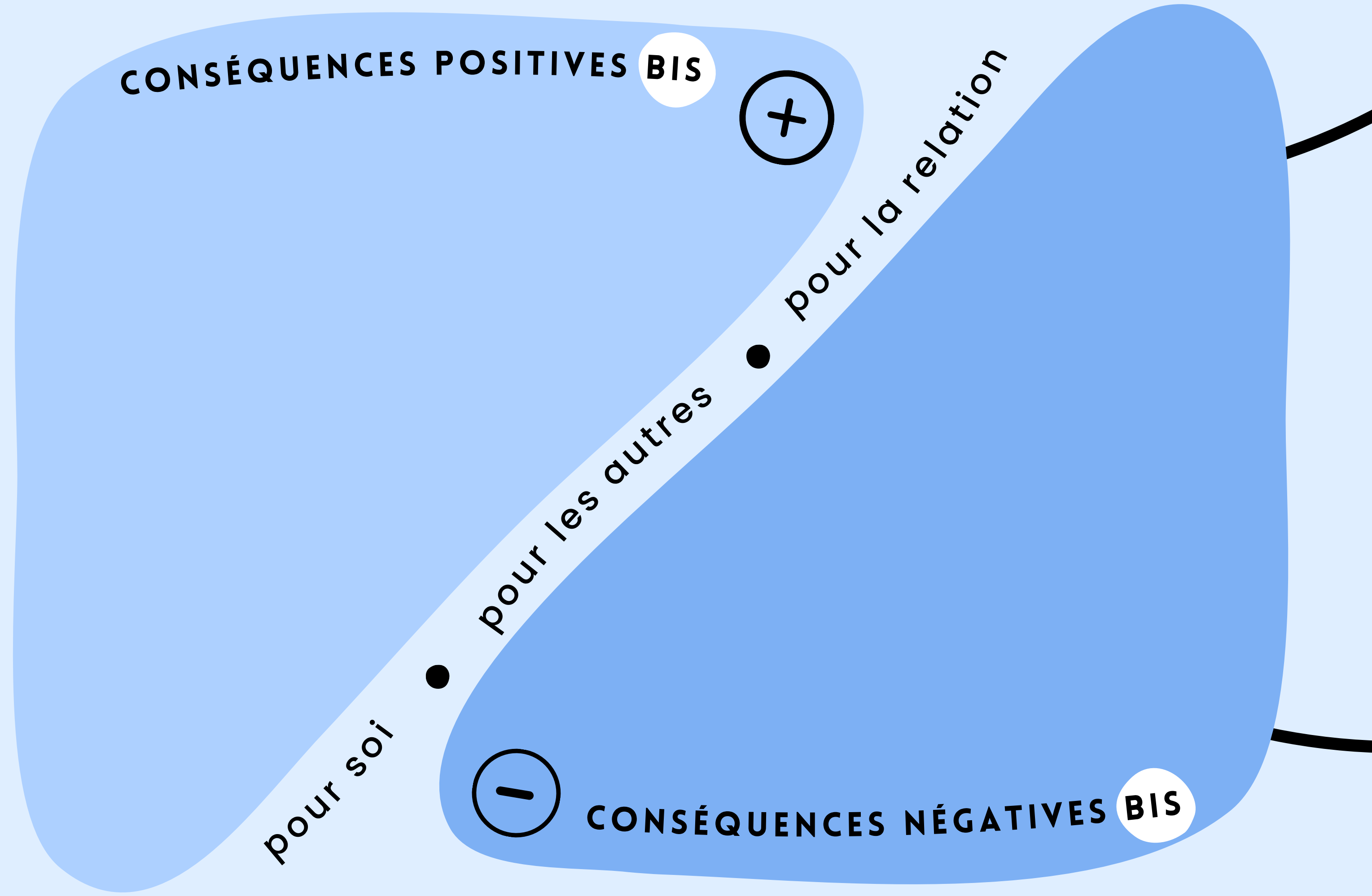

STRATÉGIES

	Avant	Pendant	Après
1.	☐	☐	☐
2.	☐	☐	☐
3.	☐	☐	☐
4.	☐	☐	☐
5.	☐	☐	☐
6.	☐	☐	☐

STRATEST

- ☐ est satisfait-e ____________________
- ☐ est satisfait-e ____________________
- ☐ et s'entendent mieux ____________________
- ☐ Le problème est résolu ____________________
- ☐ La situation est apaisée ____________________
- ☐ Autre (préciser) ____________________

BILAN

stratégies validées ✔

✘ retour case stratégies

① STRATAIDE

Nota Bene : Je peux **combiner autant de stratégies** que je le veux !

PARLER EN JE
Je pense, **j'**ai l'impression,
je me sens...

DIRE MES ÉMOTIONS, BESOINS
Je ressens/ me sens... **car**...
J'ai **besoin** de... / que...

NON-VERBAL
Utiliser le ton de la voix, les gestes

FAIRE UNE DEMANDE
en décrivant **précisément l'action** que j'attends

DEMANDER PARDON
Je suis désolé-e de...

ENQUÊTE
J'aimerais mieux comprendre...
Est-ce que **tu peux m'expliquer**...?
(**Quand**) est-ce que **tu serais disponible** pour parler de... ?

PROPOSER
Je te propose que...
Que penses-tu que... ?

REMERCIER
Merci d'avoir écouté, d'avoir accepté de faire un effort

EMPATHIE
Je comprends que...
Je suis désolée que tu...

RÉAGIR DIFFÉREMMENT
quoi ? comment ?

FAIRE UN PAS VERS L'AUTRE
Une **action** pour
se rapprocher de l'autre

AIDER L'AUTRE
Une **action** pour **rendre service**, se racheter,
réparer une faute ou une erreur

SE CALMER
S'éloigner, s'isoler, respirer lentement

INDISPONIBILITÉ
Je ne me sens pas disponible pour en parler maintenant

MÉDIATEUR
Demander l'avis ou l'intervention de quelqu'un

---

② STRATAIDE SPÉCIAL CONFLITS

CAUSE DU PROBLÈME
- selon α
- selon Ω

LISTE DES SOLUTIONS POSSIBLES
- pour α
- pour Ω

de la meilleure à la moins bonne

EFFORTS
- que α
- et Ω

sont prêt-e à faire

PREMIER PAS
- que α
- et Ω

sont prêt-e-s à faire l'un-e vers l'autre

COMPROMIS FINAL
la meilleure solution,
à la fois pour α et pour Ω,
inspirée des idées de chacun

STRATEST

STRATÉGIES BIS Ω

	Avant	Pendant	Après
1b.	☐	☐	☐
2b.	☐	☐	☐
3b.	☐	☐	☐
4b.	☐	☐	☐
5b.	☐	☐	☐
6b.	☐	☐	☐

STRATAIDE +

A LA RECHERCHE DU POSITIF

Qu'est-ce que j'aime bien chez l'autre ?

- comme qualités
- comme actions

lesquelles ?

PROPOSER

une sortie,
une activité…
à partager

quoi ? à qui ?

ACT KIND

Proposer mon aide

Trouver un acte de gentillesse

quoi ?
comment ? à qui ?

FAIRE UN COMPLIMENT

Dire à quelqu'un ce que j'aime chez lui/elle (être précis-e)
une action, une réussite, son apparence…

qui ? quoi ?

UTILISER MON CORPS

avec un sourire,
une expression,
ma voix,
un geste…

MERCI

Remercier l'autre pour quelque chose qu'iel a dit ou fait,
qui m'a aidé-e, touché-e…

qui ? pour quoi ?
